# Spontaneous Resolution of Parastomal Gallbladder Herniation After Attempted Surgical Intervention: A Case Report and Review of the Literature

**DOI:** 10.7759/cureus.37355

**Published:** 2023-04-10

**Authors:** Nahstajia Pinnock, Aksal Vashi, Jordan W Marsh, Mamadi Papus Keita, Allyn Checovich

**Affiliations:** 1 General Surgery, Carle Health, Urbana, USA; 2 General Surgery, Carle Illinois College of Medicine, Urbana, USA

**Keywords:** hernia repair, cholecystectomy, cholelithiasis, gallbladder, parastomal hernia

## Abstract

Cholecystic parastomal herniation is a rare condition that has only been documented 16 times in the literature. We present a case report and literature review of cholecystic parastomal herniation managed with diagnostic laparoscopy without cholecystectomy or hernia repair. Furthermore, we assess the demographics, presentation, stoma types, and management of cholecystic parastomal hernias across all documented cases.

## Introduction

There are approximately 100,000 operations for stoma creation performed in the United States each year [1]. An estimated 725,000 to 1 million people currently live with a stoma or continent diversion in the United States alone [1]. Parastomal hernias are a subset of incisional hernias that arise through a fascial defect between the enterostomy and abdominal wall. Parastomal hernias are well-known complications of stoma creation often involving protrusion of intra-abdominal contents such as omentum, small bowel, or colon. Incidence rates can be as high as 65% [2]. Herniation of the gallbladder into a parastomal hernia represents a rarely encountered and described entity in the literature. Our case represents a rarer entity in which a large gallstone protruded into the parastomal hernia, and is the second described case of such an occurrence in the literature.

## Case presentation

The patient was a 59-year-old female with a past medical history significant for type two diabetes, hyperlipidemia, asthma, and Crohn's disease who underwent an exploratory laparotomy, completion proctectomy with end ileostomy for large bowel obstruction secondary to a stricture of the rectum 13 years prior to presentation to the hospital. The patient had undergone two prior colon resections with primary anastomoses for Crohn's colitis which were complicated by anastomotic strictures and refractory Crohn's colitis despite maximal maintenance therapy. She presented to the hospital with complaints of right upper quadrant abdominal pain, nausea, and vomiting that started one week prior to presentation to the hospital. During this time, she continued to produce a stable amount of liquid and gas output from her stoma. She also complained of subjective fevers. She initially attributed her symptoms to starting a new oral anti-hyperglycemic medication which she had taken the day prior to the onset of symptoms.

On physical examination, palpation of her right upper quadrant elicited a significant amount of tenderness. Her ileostomy, located in the right mid-abdomen, was pink and productive of liquid stool without signs of ischemia. Upon digital stoma examination of the ileostomy, a finger was easily passed beyond the level of the fascia and there were no palpable masses. The patient was hemodynamically stable on presentation. Laboratory values were notable for a leukocytosis of 11.45 and a significantly elevated creatinine level of 5.22 mg/dL (reference range 0.55 - 1.02 mg/dL) above the patient’s baseline representing acute kidney injury (AKI) likely secondary to dehydration from repeated vomiting and poor oral intake. The patient was admitted for fluid resuscitation.

**Figure 1:**
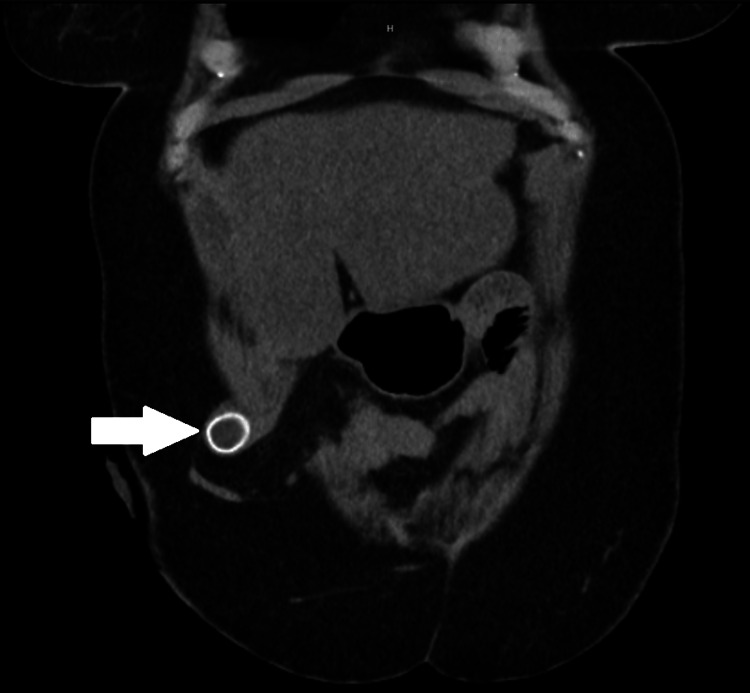
CT Abdomen and Pelvis with coronal view of parastomal gallbladder herniation White arrow demonstrating gallbladder with 2-cm gallstone herniating through parastomal hernia

**Figure 2:**
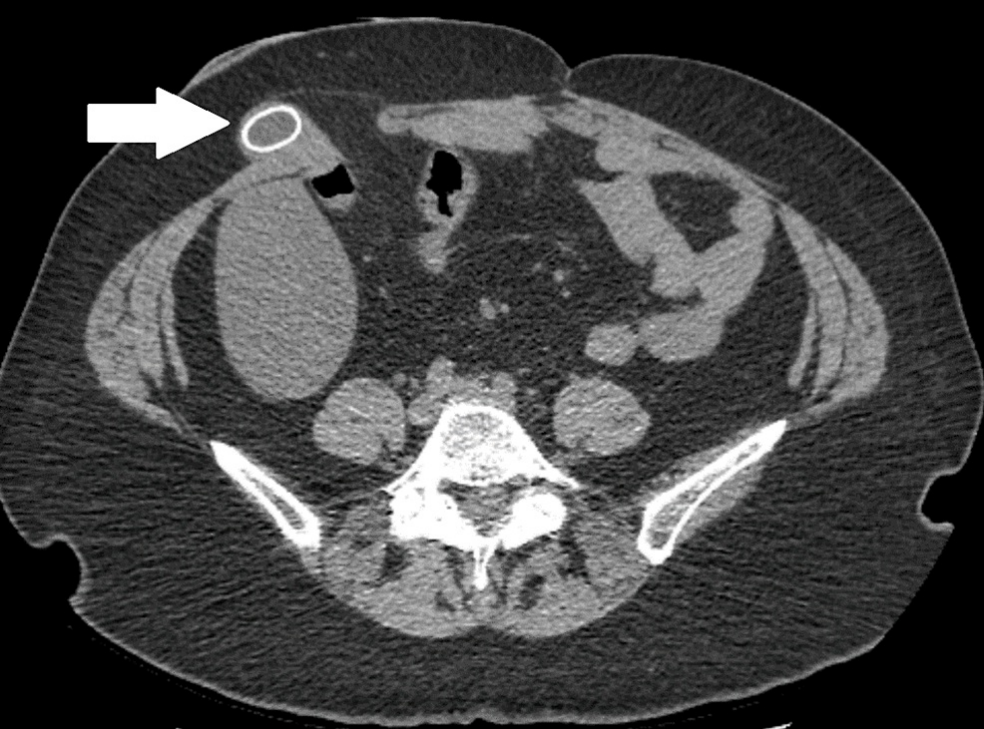
CT Abdomen and Pelvis with axial view of gallbladder herniation into parastomal hernia White arrow demonstrating gallbladder with 2-cm gallstone herniating through parastomal hernia

A non-contrast computed tomography (CT) scan of the abdomen and pelvis was obtained demonstrating a small parastomal hernia containing the gallbladder fundus and a 2-cm stone within the fundus of the gallbladder (Figures 1, 2). There was also mild gallbladder wall thickening noted but no pericholecystic fluid therefore findings were equivocal for acute cholecystitis. Initial supportive measures were instituted with nothing per os (NPO) for bowel rest. Per our discussion with the patient during the informed consent process, the patient expressed concern about the possible complications that could arise during the operation for parastomal hernia repair such as inadvertent enterotomy potentially necessitating revision of the end ileostomy. After an extensive conversation with the patient regarding the risks and benefits of having the parastomal hernia repaired during this operation the patient declined to pursue parastomal hernia repair, electing to only pursue reduction of the gallbladder and cholecystectomy if warranted at the time of diagnostic laparoscopy.

**Figure 3:**
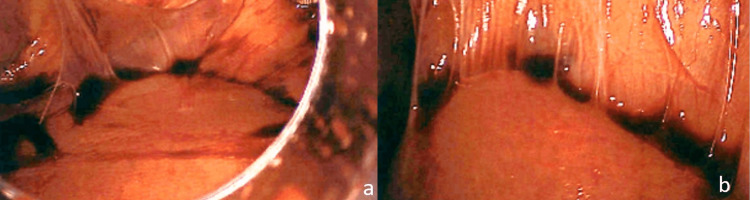
Extensive intra-abdominal adhesions and spontaneous reduction of parastomal gallbladder herniation a. Intra-abdominal adhesions b. Intra-abdominal adhesions to the liver

On hospital day two, the patient was taken to the operating room for diagnostic laparoscopy due to the concern for incarceration of the gallbladder fundus within the parastomal hernia. A 12-mm subxiphoid vertical midline incision was made, and an open Hassan technique was used to gain access to the abdominal cavity. Upon inspection, the view of the abdominal cavity was nearly entirely obscured by adhesions (Figure 3). The parastomal hernia site, however, could be visualized, and the gallbladder no longer appeared to be entrapped within the hernia. Thus, it appeared the hernia contents had been reduced. At this point the decision was made to abort the operation given the extensive adhesive disease and the increased likelihood of inadvertent enterotomy if further manipulation of the bowel were pursued.

The patient’s abdominal pain improved over the subsequent days. Her diet gradually advanced from clear liquids to a diabetic diet which she tolerated. Her ileostomy continued to be productive of gas and liquid effluent without concern for obstruction. Her creatinine returned to baseline levels with adequate fluid resuscitation and oral intake. The patient was discharged home on hospital day four and post-operative day two.

## Discussion

While cholecystic parastomal herniation is uncommon, we identified 16 previously reported cases of its management (Table 1) [3-18]. Including our case, the mean age of cholecystic parastomal herniation presentation is 72.8 years of age with a female predominance of 88%. As discussed in Moeckli et al. 2020, the advanced age of presentation can be directly correlated with decreasing connective tissue elasticity in elderly populations [8]. 

**Table 1:** Previously documented parastomal gallbladder herniation cases

Year	Age	Sex	Type of Stoma	Diagnostic modality	Presentation	Treatment	Outcome
2022 [3]	87	F	Ileal conduit	Computed tomography	Symptomatic, right upper quadrant pain with worsening parastomal swelling	Surgical, open cholecystectomy without hernia repair	Discharged after one month in patient rehab after postoperative myocardial infarction
2021 [4]	71	F	End ileostomy	Computed tomography	Symptomatic, increasing abdominal pain	Conservative, analgesia and manual reduction	Discharged day after manual reduction; labs normalized 10 days later
2021 [5]	65	F	Loop ileostomy	Computed tomography	Asymptomatic, painless parastomal bulge	Surgical, ileostomy annulation with end colostomy, no cholecystectomy	Discharged on post-operative day 4; no complications at six months
2021 [6]	87	F	End Ileostomy	Computed tomography	Symptomatic, increasing abdominal pain at parastomal hernia	Surgical, open cholecystectomy	Discharged on post-operative day 11. Doing well at six month follow-up.
2020 [7]	63	F	End ileostomy	Computed tomography	Symptomatic, fever, vomiting, abdominal pain	Failed conservative; Surgical, cholecystectomy with biological porcine dermal collagen mesh	Readmitted for worsening abdominal pain and fever; no recurrence at 5 month follow up
2020 [8]	69	F	End ileostomy	Computed tomography	Symptomatic, parastomal swelling, nausea, pain	Surgical, open cholecystectomy	Discharged POD 34; no recurrence at 4 years
2019 [9]	75	F	End ileostomy	Computed tomography	Symptomatic, increasing abdominal pain, incarcerated hernia	Surgical, no cholecystectomy but reinforcement of PSH with synthetic on-lay mesh	Discharged on POD 5 no complications at 6 months
2018 [10]	63	F	End transverse colostomy	Computed tomography	Asymptomatic, painless parastomal bulge	Surgical, ostomy takedown, cholecystectomy, abdominal wall reconstruction with on-lay bio prosthetic mesh	No recurrence at follow-up
2017 [11]	89	M	Diverting loop ileostomy	Computed tomography	Symptomatic, burning around stoma and small bowel obstruction	Conservative due to comorbidities, antibiotics, nasogastric tube	Discharged on hospital day 5
2017 [12]	88	F	Transverse loop colostomy	Computed tomography	Symptomatic, fever, abdominal pain	Conservative due to comorbidities, antibiotics, nasogastric tube	Discharged on hospital day 7
2017 [13]	50	F	Right hypochondrium diverting colostomy	Computed tomography	Symptomatic, abdominal pain and vomiting	Surgical, Right subcostal laparotomy, cholecystectomy and primary closure of the defect on the inner face without mesh	Discharged well, follow up not reported
2015 [14]	85	F	Ileal conduit	Computed tomography	Symptomatic, increasing pain at ileal conduit	Surgical, open cholecystectomy without closure of hernia defect	Discharged POD 5, well at 1 month visit
2013 [15]	76	M	End ileostomy	Computed tomography	Symptomatic, abdominal pain	Surgical, open cholecystectomy	Discharged POD 5
2010 [19]	74	F	End ileostomy	Surgical exploration	Symptomatic, tender parastomal hernia	Surgical, open cholecystectomy, pre-peritoneal mesh repair of hernia	Recurrence of parastomal hernia at 16 months
2005 [16]	63	F	Transverse colostomy	Computed tomography	Symptomatic, nausea, abdominal pain	Conservative due to comorbidities	Discharged on hospital day 2, asymptomatic at 16 months
2005 [17]	73	F	Ileal conduit	Surgical exploration	Symptomatic, abdominal pain, hernia incarceration	Surgical, open cholecystectomy without closure of hernia	Not applicable

Additionally, based on the observed cases, the type of stoma seems to have an impact on cholecystic parastomal herniation prevalence. Approximately 13 out of 17 (76%) patients in all reported cases had an ileal stoma or ileal conduit. Among these, eight out of 13 (61.5%) were associated with end ileostomies. The higher frequency of cholecystic parastomal herniation in conjunction with ileal stomas could be in part due to ileal stomas being located in the right hemiabdomen close to the gallbladder. Furthermore, gallbladders with a long mesentery may have a propensity for herniation into nearby abdominal wall defects.

Surgical management of parastomal hernias remains a challenge despite the high recurrence rates (37-76%) [18,20]. Techniques such as laparoscopic parastomal hernia repair with mesh utilizing the Sugarbaker technique show low infection and recurrence rates [18]. The rarity of parastomal hernias with gallbladder herniation further adds to the lack of clarity surrounding the management of these rare parastomal hernia complications. Of the 17 known cases, 13 were managed with surgical intervention: 10 involving cholecystectomy and five involving parastomal hernia repair (three biological mesh repairs, one synthetic mesh repair, and one primary repair without mesh). The only known hernia recurrence amongst all cases occurred in Rashid et al. 20 months after pre-peritoneal mesh repair [16]. 

Of the 17 reported cases of gallbladder herniation into a parastomal hernia described in our literature review, five were managed non-operatively. Of these five cases, there was one recurrence described by Guo et al. Initial conservative measures were taken for this patient per patient/family wishes to limit surgery, however, the patient ultimately required surgical intervention with cholecystectomy and parastomal hernia repair with biologic mesh placement [4,7,11,12,17]. Three of the conservatively managed cases pursued non-operative management due to patient comorbidities. One case was managed conservatively after resolution of symptoms from manual reduction of the hernia. Our patient presented with symptomatic cholelithiasis consistent with CT imaging, physical exam, and laboratory findings. The herniation of the gallbladder fundus associated with the large 2-cm gallstone heightened concerns for possible incarceration with resultant ischemia, therefore it was deemed necessary to perform a diagnostic laparoscopy with consideration for concomitant cholecystectomy and parastomal hernia repair.

This is the first case to describe an attempt at minimally invasive approach without cholecystectomy or hernia repair. Previous cases demonstrate that the presentation and related gallbladder pathologies involved with parastomal gallbladder herniations vary widely. Some note overlying skin changes associated with parastomal herniation of the gallbladder, cholecystic perforation, significant cholelithiasis burden, cholecystic incarceration/strangulation, and associated acute cholecystitis. Our patient demonstrated resolution of symptoms with spontaneous reduction of the gallbladder herniation into parastomal and a return to her baseline status of health. 

## Conclusions

While there is no standardized approach to the management of parastomal gallbladder herniation, the 17 known cases demonstrate that the most appropriate intervention should be based on the individual patient and their underlying pathology. Intervention should not be solely focused on the herniation of the gallbladder itself. Our case represents an attempt at surgical intervention that was aborted due to the patient’s extensive adhesive burden precluding safe operative intervention. Furthermore, the parastomal gallbladder herniation spontaneously resolved after induction of general anesthesia. This case report contributes to the scarce but growing body of literature regarding this rare entity and its elusive management algorithm.
